# Utilizing Spectral, Structural and Textural Features for Estimating Oat Above-Ground Biomass Using UAV-Based Multispectral Data and Machine Learning

**DOI:** 10.3390/s23249708

**Published:** 2023-12-08

**Authors:** Rakshya Dhakal, Maitiniyazi Maimaitijiang, Jiyul Chang, Melanie Caffe

**Affiliations:** 1 Plant Breeding Graduate Program, University of Florida, Gainesville, FL 32608, USA; rakshya.dhakal@ufl.edu; 2 Department of Geography and Geospatial Sciences, South Dakota State University, Brookings, SD 57007, USA; maitiniyazi.maimaitijiang@sdstate.edu; 3 Department of Agronomy, Horticulture and Plant Science, South Dakota State University, Brookings, SD 57007, USA; jiyul.chang@sdstate.edu

**Keywords:** biomass, machine learning, multispectral imagery, oat, remote sensing, UAV

## Abstract

Accurate and timely monitoring of biomass in breeding nurseries is essential for evaluating plant performance and selecting superior genotypes. Traditional methods for phenotyping above-ground biomass in field conditions requires significant time, cost, and labor. Unmanned Aerial Vehicles (UAVs) offer a rapid and non-destructive approach for phenotyping multiple field plots at a low cost. While Vegetation Indices (VIs) extracted from remote sensing imagery have been widely employed for biomass estimation, they mainly capture spectral information and disregard the 3D canopy structure and spatial pixel relationships. Addressing these limitations, this study, conducted in 2020 and 2021, aimed to explore the potential of integrating UAV multispectral imagery-derived canopy spectral, structural, and textural features with machine learning algorithms for accurate oat biomass estimation. Six oat genotypes planted at two seeding rates were evaluated in two South Dakota locations at multiple growth stages. Plot-level canopy spectral, structural, and textural features were extracted from the multispectral imagery and used as input variables for three machine learning models: Partial Least Squares Regression (PLSR), Support Vector Regression (SVR), and Random Forest Regression (RFR). The results showed that (1) in addition to canopy spectral features, canopy structural and textural features are also important indicators for oat biomass estimation; (2) combining spectral, structural, and textural features significantly improved biomass estimation accuracy over using a single feature type; (3) machine learning algorithms showed good predictive ability with slightly better estimation accuracy shown by RFR (R^2^ = 0.926 and relative root mean square error (RMSE%) = 15.97%). This study demonstrated the benefits of UAV imagery-based multi-feature fusion using machine learning for above-ground biomass estimation in oat breeding nurseries, holding promise for enhancing the efficiency of oat breeding through UAV-based phenotyping and crop management practices.

## 1. Introduction

Oat (*Avena sativa* L.) is among the most widely cultivated small grains and is primarily grown for forage and feed grain. It is considered as a superior forage crop because of its fine stem [1], high dry matter content, and presence of digestible fibers in the leaves contributing to high palatability [2,3]. In comparison to perennial forage crops such as alfalfa, oat provides a quick supply of high-quality forage as an annual crop; it is fairly easy to establish and harvest, and it has low production and management cost [4]. Improving forage yield and quality of oat varieties is a key objective in oat breeding programs across the US. Forage yield, which corresponds to above-ground biomass, is a complex trait controlled by multiple genes [5] and is affected by the environment to varying degrees. Therefore, multi-environment large scale trials are often set up in breeding programs to evaluate trait stability across environments.

Conventional phenotyping methods for quantifying above-ground biomass often require manual measurement of biomass via cutting, weighing, and drying a sub-sample for moisture estimation. This process is highly tedious, labor-intensive, costly, and destructive [6,7], and limits the extent of phenotyping since it is not operationally feasible for large number of genotypes over multiple environments [8]. However, large scale multi-environment trials are crucial to measure the extent of genotype-by-environment interactions. Remote sensing technologies, including satellites, aircraft, Unmanned Aerial Vehicles (UAVs), Unmanned Ground Vehicles (UGVs), and handheld instruments and sensors, have been used as important tools for crop monitoring, and phenotyping. Aircraft and satellite-based observations cover large areas, but the data often suffer from coarse spatial resolution, the effect of clouds, and low temporal resolution [9,10]. Handheld and UGV-based phenotyping are often inefficient, time consuming, and may destroy crops and fields, limiting their applicability on a large scale [11]. In contrast, UAV-based remote sensing is gaining popularity in plant phenotyping, especially crop biomass estimation applications due to its cost effectiveness, as well as efficient and non-destructive nature of data collection [12]. Additionally, UAVs enable flexible and high resolution image acquisition [13], can avoid cloud cover disturbances, and are excellent for extracting plot-level information from large fields. They can be equipped with different sensors, making them an effective field phenotyping tool [14,15]. RGB [16,17], multispectral [18], and hyperspectral images acquired from UAVs provide improved spectral, spatial, and temporal resolution, in comparison to images acquired from satellite and airborne platforms. High throughput phenotyping using UAVs has been reported for estimation of ground cover [19,20], nitrogen concentration [21,22], and grain yield [23,24,25]. Similarly, UAVs have been also employed for plant biomass estimation in alfalfa [5], grass swards [26], tomato [27], winter wheat [7], sorghum [28], black oat [29], soybean [6], and barley [30].

Canopy spectral, structural, textural, and thermal features extracted from UAV-based imagery can be used to estimate plant biomass. Spectral features, such as Vegetation Indices (VIs), have been commonly employed to estimate biomass in various crops, including winter wheat [20], barley [31], rice [32], and maize [33]. However, spectral features can present limitations [34]. For example, the Normalized Difference Vegetation Index (NDVI) is a widely used VI that has a tendency of attaining asymptotic saturation once it reaches a certain canopy density [12,34]. They are often easily affected by soil background and atmospheric effects [35,36]. Moreover, canopy spectral features such as VIs are unable to capture and explain the complex three-dimensional (3D) characteristics of the canopy structure.

Canopy structural features, such as canopy height and vegetation fraction, offer a better representation of the 3D canopy structure and geometric properties. Differences in canopy height reflect the health and vigor of crops and thus, canopy height features are found to be greatly correlated with biomass either used separately or in conjunction with spectral features. Many studies have utilized 3D canopy structural features to estimate biomass in a variety of agricultural crops such as soybean [6], black oat [29], maize [37,38], winter wheat [7,39] and barley [30]. Canopy height often can be derived from photogrammetry-based or Light Detection and Ranging (LIDAR) point clouds. Bendig et al. [30] reported strong correlation of barley biomass with canopy height derived from photogrammetry-based point clouds. Combining canopy spectral and structural features have also shown great potential in crop biomass estimation in many crops [7,26,40]. 

Canopy height and density vary as plants mature, thus, characterizing spatial changes in the plant canopy is very helpful. One of the limitations of spectral indices is that they fail to capture the spatial variability of the pixel intensity level between neighboring pixels within an image [41]. Canopy textural features, on the other hand, offer valuable insights into the spatial distribution and patterns of pixel intensities in an image. They enable the assessment of changes in pixel values among neighboring pixels within a defined analysis window [42,43]. Canopy textural features are often utilized to smoothen the differences in canopy structure and geometric features [12], and to lessen the interference of background [43]. These features have found widespread application in image classification [44,45], and in forest biomass estimation [46,47], yet their potential in agricultural crop biomass estimation is less explored. Few studies have examined the potential of textural features and their incorporation with VIs for biomass estimation in agricultural crops [48,49]. Liu et. al. [49] found that the incorporation of textural features derived from multispectral imagery reduced the RMSE by 7.3–15.7% when estimating winter oilseed biomass. Similar results were reported by Zheng et al. [48] in their study estimating rice biomass, where the inclusion of textural features into multispectral VIs exhibited improved results. 

A combination of canopy structural and textural features with the spectral features derived from UAV images has the potential to deliver better estimations of biomass and crop grain yield than using a single type of features [12,49,50,51]. To the best of our knowledge, no prior studies have explored the potential of combining canopy textural features with multispectral VIs and canopy height features for oat biomass estimation. In recent years, many studies have evaluated statistical and machine learning (ML) based regression techniques for estimation of biophysical traits in a variety of crops like winter wheat [7,52], potato [16,50], maize [38], etc. Biomass growth typically follows a complex, non-linear pattern and, therefore, is hard to effectively model with linear statistical regression techniques [8,53]. ML algorithms such as Random Forest, Support Vector Machine, Gradient Boosting model, Neural Networks, etc. are gaining popularity nowadays in remote sensing-based biomass estimation due to their ability to model complex non-linear relationships between crop biomass and remote sensing variables [41,54]. Partial Least Squares Regression (PLSR), Random Forest Regression (RFR), and Support Vector Regression (SVR) are being extensively used for the purpose of crop biomass estimation. Fu et al. [55] highlighted the flexibility and efficiency of using PLSR-based modelling to predict biomass of winter wheat. Similarly, Wang et al. [36] reported that PLS and RF regressions perform well to estimate biomass of winter wheat at multiple growth stages. SVR has been found to adapt very well with complex data and can be effectively used in biomass estimation studies [56]. Sharma et al. [57] also emphasized the benefits of using PLS, SVR and RF regression for oat biomass estimation. The combination of canopy spectral, structural, and textural features derived from UAV-based multispectral imagery to predict biomass in oats using ML algorithms has not been explored. This study aims to examine the effectiveness of combining canopy textural and structural features with multispectral VIs in the estimation of oat biomass. The specific objectives of this study were to (i) evaluate the contributions of canopy structural and textural features in biomass estimation; (ii) examine whether fusion of multiple features (canopy spectral, structural, and textural) improve the accuracy of biomass estimation models for oats; and (iii) compare the performance of different ML models for oat biomass estimation.

## 2. Materials and Methods

### 2.1. Test Site and Field Data Acquision

**Figure 1:**
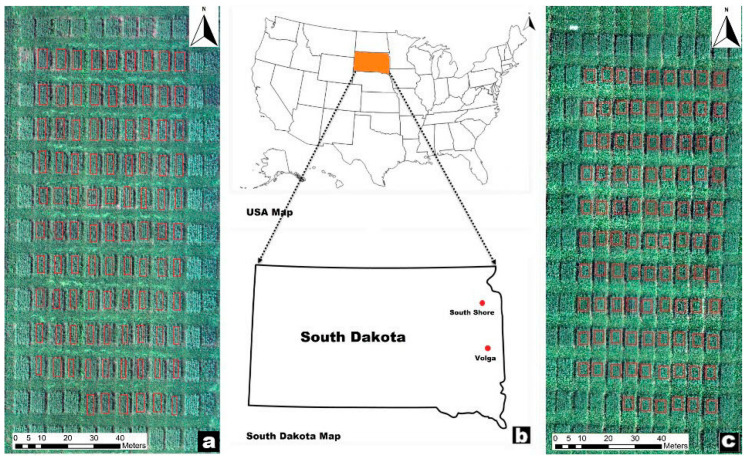
Locations of the testing sites (**b**) and imagery of the experimental plots at each sites in 2021 ((**a**) South Shore and (**c**) Volga).

A forage trial was planted at two sites in South Dakota (Volga and South Shore) in 2020 and 2021 (Figure 1). The trial included six oat genotypes (Jerry, Rushmore, Warrior, SD150081, SD120665, and SD150012) were planted at two seeding rates (approximately 150 and 300 seeds/m^2^) at a depth of approximately 0.038 m. Each plot was 1.524 m by 1.219 m. The trials were managed by using recommended agronomic practices for proper growth and yield. 

The experimental design consisted of a completely randomized block design with 8 replicates. Replications 1, 2, and 3 were harvested at booting, replications 4, 5, and 6 were harvested at heading, and replications 7 and 8 were harvested at the milk stage (Table 1). The multiple growth stage harvests and planting at two seeding rates ensured a wider range of obtained biomass. 

Throughout the growing season, oat growth was continuously monitored. On the same days as harvesting, field measurements of canopy height and biomass sampling were conducted to gather accurate and reliable data. The canopy height was measured using a representative plant selected to reflect the average height of all plants within a plot. This measurement, taken just prior to harvesting, recorded the distance from the soil to the tip of the chosen plant’s panicle.

To obtain the above-ground fresh biomass, the plots were carefully cut close to the ground using a Jari mower. During the harvesting process, the bordering rows on both sides of the plot were excluded to ensure precise biomass sampling. A small subsample of the biomass was collected, weighed, and dried in an oven set at 75 °C until a stable weight was achieved. The dry matter content was determined by dividing the weight of the dried subsample by the weight of the corresponding fresh subsample and multiplying the result by 100%. The dry biomass yield was calculated by multiplying the dry matter content by the fresh weight of the biomass, expressed in kilogram per hectare.

### 2.2. UAV Data Acquisition and Image Preprocessing

**Figure 2:**
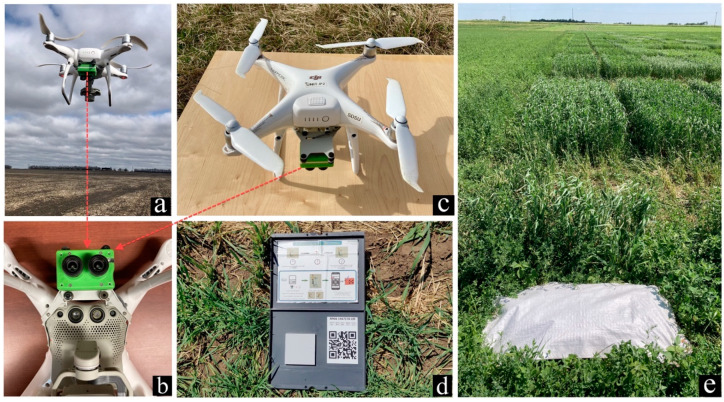
UAV systems and their setup in the field. (**a**) DJI Phantom 4 pro and attached Sentera double 4K sensor; (**b**) Sentera double 4K sensor (zoomed view); (**c**) DJI Phantom 4 pro (top view); (**d**) Micasense calibrated panel; (**e**) white poly tarps as ground control points (GCPs).

Aerial images were collected on the day of biomass harvest with a DJI phantom 4 pro UAV (SZ DJI Technology Co., Shenzhen, China) (Figure 2c) equipped with a Multispectral Double 4 K camera (Sentera Inc., Minneapolis, MN, USA) (Figure 2b). The camera has a 12.3-megapixel BSI CMOS Sony Exmor R™ IMX377 Sensor. It can capture five precise spectral light bands: blue, green, red, red-edge, and near-infrared (NIR). The central wavelength and full width at half maximum (FWHM) bandwidth of each spectral bands is presented in Table 2. 

The UAV flights were conducted on sunny, cloud-free days with minimal sun shadow and low wind speeds (wind gusts less than 12 miles per hour). The flights were performed at an altitude of 25 m with 80% front and side overlap. Four white woven polypropylene bags of size 0.35 m by 0.66 m, fixed permanently in the ground throughout the growing period, were placed in the corners of the fields and used as Ground Control Points (GCPs) for georeferencing purpose (Figure 2e). The geographic coordinates of these GCPs were measured using Mesa ^®^ Rugged Tablets from Juniper Systems (Juniper Systems, Logan, UT, USA). Additionally, a calibration panel (Micasense Inc., Seattle, WA, USA) with known reflectance factors was used for radiometric correction purposes (Figure 2d).

Digital Surface Model (DSM) and orthomosaic imagery were generated using Pix4DMapper software v 4.8.0 (Pix4D S.A., Prilly, Switzerland). The process involved three main steps: initial processing for key point computation and image matching, radiometric calibration for converting raw digital numbers to reflectance values, and DSM generation for surface elevation representation. Georeferencing was performed using GNSS coordinates of four Ground Control Points (GCPs) recorded in QGIS software v 3.20 (QGIS Development Team, Open Source Geospatial Foundation). Georeferenced images were saved in a TIFF format and exported to ArcMap software for creating a 5-band orthomosaic and for spectral indices extraction and DTM generation.

### 2.3. Features Extraction

Following image preprocessing, various features were extracted to be used as input variables for oat biomass estimation. These included canopy spectral indices such as VIs, canopy structural features such as plant height, and canopy textural features. For each plot, a rectangular region of interest (ROI) or plot was defined on the images, and a polygon shapefile was created using ArcMap software (Redlands, CA, USA). Plot-level statistics were obtained by averaging pixel values from spectral, structural, and textural raster layers using the zonal statistics tool. 

#### 2.3.1. Spectral Features 

Ten different VIs were calculated using the reflectance bands (red, green, blue, NIR, and red edge) obtained from the multispectral sensor (Table 3). These VIs were categorized into seven multispectral VIs (1–7 in Table 3) and three true-color VIs (8–10 in Table 3). The selection of these VIs was guided by their ability to correlate well with biomass and their sensitivity to changes in greenness and vegetation vigor, as observed in previous studies [20,57,58,59]. 

#### 2.3.2. Structural Features

**Figure 3:**
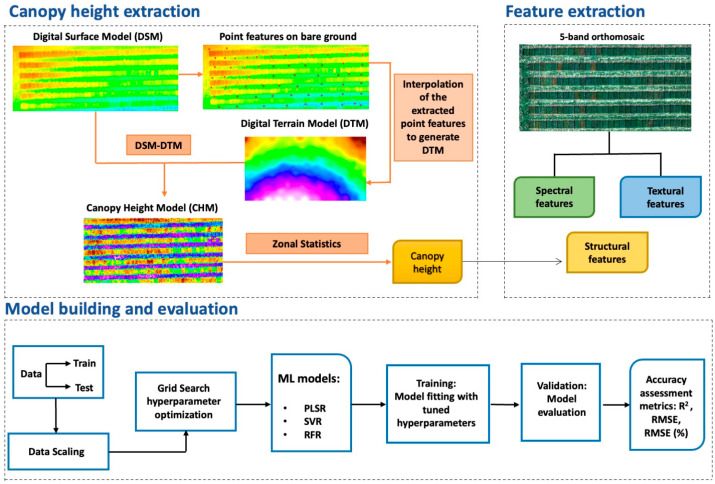
Workflow of feature extraction and model development for biomass estimation in this study.

Height features were derived through digital photogrammetry-based point clouds. The Digital Surface Model (DSM) obtained from Pix4DMapper software was imported into ArcGIS software. The DSM represents the ground’s structures, encompassing both bare earth and canopy. Since we were not able to collect preplant bare soil UAV imagery to generate Digital Terrain Model (DTM), which represents the soil surface elevation, thus, bare surface points were selected from the DSM imagery and interpolated using the Inverse Distance Weighting (IDW) method, resulting in a raster layer representing ground surface elevation. The Canopy Height Model (CHM) was obtained by subtracting the DTM from the DSM, capturing the height of the vegetation (CHM = DSM − DTM). Figure 3 provides a comprehensive illustration of the canopy height extraction process.

The CHM and shapefile were subsequently exported to ArcGIS software to calculate height features for each plot, including average height (H_mean_), maximum height (H_max_), minimum height (H_min_), median height (H_median_), height standard deviation (H_std_), height 90% percentile (H_p90_), height 93% percentile (H_p93_), and height 95% percentile (H_p95_). (Table 4).

#### 2.3.3. Textural Features

Textural features for the plots in each image were computed using the Gray Level Co-occurrence Matrix (GLCM) texture algorithm, introduced by Haralick et al. [70]. The GLCM provides information on the spatial relationship of pixel pairs in an image and is one of the widely used image textural features in remote sensing. Using the ENVI 5.6.1 software, different textural features were calculated for each of the five bands, including variance (VAR), mean (ME) homogeneity (HOM), dissimilarity (DIS) contrast (CON), entropy (ENT), angular second moment (ASE), and correlation (COR). A 3 × 3 moving window was set for the calculations. Further details about these textural features can be found in [71]. Table 5 presents the list of GLCM textural features used in this study. To denote each extracted texture, they are prefixed with either “R-”, “G-”, “B-”, “NIR-” and “Red edge-” to denote the GLCM-based textures for the five bands (e.g., R-ME denotes the mean of the red band).

### 2.4. Statistical Analysis

#### 2.4.1. Data Preprocessing and Feature Selection

All data points including UAV imagery features and corresponding biomass ground truth values from the 2 years and two fields were combined for further analysis and modeling (Table 6). The dataset had ground-truth biomass and UAV imagery features obtained from all three harvests: the first three replications from the booting stage harvest, the next three from the heading stage harvest, and the final two from the milk stage harvest. The dataset was first subjected to a preliminary statistical test to check the presence of any outliers. Then, the correlation between canopy height features obtained from CHM and canopy height obtained from ground measurement (H_ref_) were calculated to investigate the accuracy and quality of UAV-based canopy height data. In addition, correlations between the spectral indices, structural features, and textural features with biomass were determined and used for feature selection. 

#### 2.4.2. Biomass Estimation Modelling

##### Machine Learning Models

Machine learning (ML) algorithms like Partial Least Squares Regression (PLSR), Random Forest Regression (RFR), and Support Vector Regression (SVR) were used to develop predictive models for oat biomass yields. The implementation of ML methods was conducted using the Scikit-learn library [72] in Python (Python version 3.9.7). 

##### Model Building and Evaluation 

A 10-fold cross-validation analysis was performed by randomly splitting all data points (n = 384) into the training dataset (70%) for calibrating the model and the testing dataset (30%) for model testing. Feature scaling was carried out before fitting our model. To find the best set of hyperparameters for each model, a hyperparameter optimization strategy, namely Grid search cross-validation, was used. 

To evaluate and compare the model performance, the coefficient of determination (R^2^), root mean square error (RMSE), and relative RMSE (RMSE%) were calculated as follows:

$$R^{2}=1-\frac{\sum_{i=1}^{n}{\left(y_{i}-\hat{y_{i}}\right)}^{2}}{\sum_{i=1}^{n}{\left(y_{i}-\bar{y}\right)}^{2}}$$

$$RMSE=\sqrt{\frac{\sum_{i=1}^{n}{\left(y_{i}-\hat{y_{i}}\right)}^{2}}{n-1}}$$

$$RMSE\%=\frac{RMSE}{\bar{y}}\times 100$$

where $y_{i}$ and $\hat{y_{i}}$ are the measured and predicted biomass yield, respectively; $\bar{y}$ is the mean of measured biomass yield; and *n* is the total number of samples in the validation set. A detailed workflow showing feature extraction (spectral, structural, and textural) and modelling using traditional ML algorithms for biomass estimation of oats is presented in Figure 3.

## 3. Results and Discussions

### 3.1. Statistical Analysis of Biomass Data 

The genotype, seeding rate, year, location, and the growth stage all affected biomass yield. Growing conditions in 2020 were more favorable than in 2021 at both locations; as a result, the biomass production was higher in 2020 compared to 2021 (Table 3). In 2021, the drought stress experienced in June reduced mean biomass production by 63.37%. The location (Volga vs. South Shore) also affected biomass production. In 2020, the highest biomass was produced in South Shore (Table 3). The severe (*Puccinia coronate* f. sp. *avenae*) infections observed on susceptible cultivars in Volga that year likely contributed to the lower biomass production at that location in comparison to South Shore. In 2021, however, higher biomass was produced in Volga compared to South Shore (Table 3). The more severe drought stress in South Shore likely affected plants more severely in comparison to those at the Volga site. Finally, as expected, we consistently observed an increase in biomass production at later growing stages. Biomass production increased by 56% between booting and milk stage in 2020 and by 112% between those two stages in 2021. Overall, in this study, a wide range of biomass was obtained, ranging from 2201.4 to 20,415.8 kg/ha (Table 3).

### 3.2. Correlation Analysis

#### 3.2.1. Relationships between Manually Measured and UAV-Estimated Canopy Height

Pearson’s correlation coefficient (r) was calculated between the manually measured and UAV-estimated canopy height. Compared to other height features, a strong correlation (r = 0.77) is found between H_p90_, H_p93_, and H_p95_ of estimated canopy height and manually measured canopy height (H_ref_) (Table 7). A similar relationship between the 90th percentile of UAV estimated canopy height and manually measured canopy height was reported in a study to predict sorghum biomass [58]. In our study, some negative values were reported for the minimum canopy height estimated from UAV imagery. This could be attributed to errors incurred during the interpolation step of DTM extraction, which added noise. While the DSM-extracted canopy height is surely a low-cost solution, there is a high chance of error in this method as it requires large bare buffer zones that are not always assured [16] and a high resolution in the extracted DTM, which is also a problem with multispectral imagery. As an alternative approach, DEM can be acquired by capturing images of the bare ground prior to plant emergence. This method would be more practical and would offer more reliable estimates of extracted canopy height, provided an adequate number of ground control points is available.

#### 3.2.2. Relationships between UAV Imagery-Extracted Features and Biomass

Pearson’s correlation coefficient (r) was calculated between biomass and UAV imagery extracted spectral, structural, and textural features (Table 8 and Table 9). Among the three different feature types, structural features are the most strongly correlated with biomass. All spectral and structural features (except H_min_) show significant positive correlations with biomass (Table 8). 

Among the spectral features, GLI shows the strongest correlation with biomass (r = 0.63). The usefulness of GLI in predicting biomass and green vegetation has been highlighted by previous studies. Taugourdeau et al. [73] found GLI to be among the most important variables when estimating herbaceous above-ground biomass in Sahelian rangelands. In another study, GLI was found to show higher sensitivity in detecting green vegetation [74]. However, other studies reported GLI to be a weak indicator for biomass estimation [75,76]. Among the NIR-based VIs, NDRE shows the strongest correlation (r = 0.49) with biomass (Table 8). NDRE is based on Red Edge spectral band, which does not suffer from the optical saturation issue and, hence, performs well even at a higher plant density [34].

Among the structural features, H_p90_ shows the strongest correlation with biomass (r = 0.74) (Table 8). The correlation between biomass and manually measured canopy height is found to be 0.88, which is higher than the correlation observed between UAV multispectral imagery-derived height features and biomass. Most of the literature has reported promising and reliable estimates of UAV imagery extracted canopy height (r ≥ 0.8) [12,58], and, therefore, strong correlations between biomass and UAV imagery extracted canopy height were reported. Nonetheless, in our study too, moderate to strong correlations are obtained between most canopy height features and biomass. These results suggest that canopy height is an important indicator of biomass as it directly reflects plant growth (i.e., biomass) and can be used to quickly estimate oat biomass. 

Textural features are not all significantly correlated with biomass (Table 9). The features with significant correlations are all negatively correlated with biomass (r = −0.1 to −0.7). The Gray Level Co-occurrence Matrix (GLCM) textural features showing the strongest negative correlation with the biomass yield is Correlation (COR) calculated on NIR Band (r = −0.7) (Table 9). Correlation measures, in general, showed higher negative correlation with biomass than any other measures (Table 9). This correlation measure characterizes the texture of an image by measuring the joint probability of the occurrence of two specified pixel pairs. Liu et al. [50] have also reported strong correlations of canopy textural features (COR, CON, HOM) calculated on RGB bands with potato biomass, suggesting that canopy textural features are suitable in predicting biomass.

### 3.3. Oat Biomass Estimation Analysis

Machine learning models PLSR, SVR, and RFR were used to predict the biomass of oats using UAV multispectral imagery-derived spectral, structural, and textural features individually and in combination. The 10 VIs calculated using five-spectral bands, along with 8 texture parameters for each band gave a total of 40 textural features but only 24 were selected based on their correlation with the biomass. So, the 10 VIs, 10 canopy height features, and 24 textural features, resulted in a total of 44 features that were used for modelling. The model testing statistics for biomass estimation are presented in Table 10.

#### 3.3.1. Spectral Feature-Based Biomass Estimation

**Figure 4:**
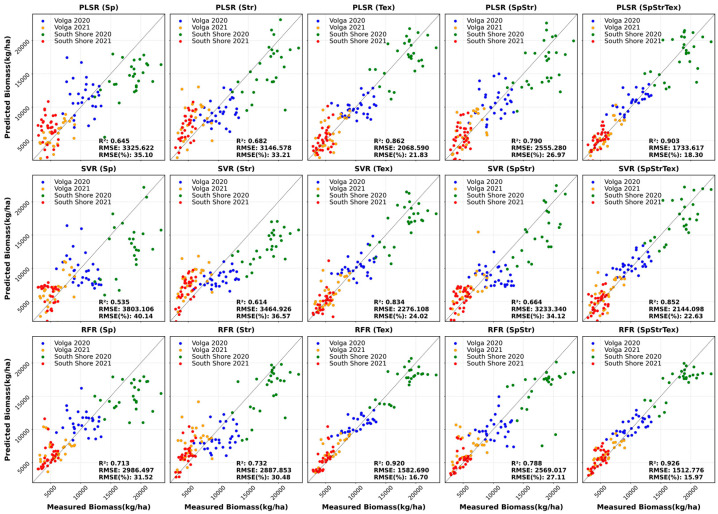
Scatter plots of measured vs. predicted oat biomass yield using different models and input features.

Variations in seeding rates, genotypic differences, and in growing environments (two locations and 2 years) led to a variation in oat canopy growth. These variations consequently led to differences in canopy spectral reflectance. Biomass estimation models were built based on PLSR, SVR, and RFR methods by using 10 VIs as input variables. The estimation accuracy obtained from three models are in the range of 0.53–0.71 (Table 10). The highest estimation accuracy (R^2^ = 0.71) and lowest estimation error (RMSE% = 35.12) was obtained from RFR modelling. The superior goodness RFR model in comparison to the other two ML models is also visible on the scatter plots as the data points are seen to be more converged towards the bisector (black dashed line) (Figure 4). The lowest accuracy (R^2^ = 0.53 and highest RMSE% = 40.14%) was yielded by SVR. Significant correlations of the VIs are seen with the biomass (Table 8) suggesting that spectral features derived from UAV-based multispectral imagery are important indicators for oat biomass estimation. Biomass estimation using canopy spectral features has been reported extensively in the literature. Many studies have shown the usefulness of using multiple VIs for biomass estimation and our results are in agreement with those previous studies. 

It is worth noting that the three models based on spectral features underestimated biomass samples with higher values (Figure 4). One reason behind this could be the optical saturation of the VIs. Similar results were also observed in winter cover crop biomass estimation [20] and soybean biomass and LAI estimation [77]. VIs that are based on NIR and red ratios (e.g., NDVI) tend to saturate at high/dense canopies [34,78], which result in poor performance of predictive models. Prabhakara et al. [20] reported that NDVI showed asymptotic saturation in the higher range of rye biomass (>1500 kg/ha). VIs are also environment and sensor specific [79]. They often do not reflect 3D canopy structure and geometrical patterns.

#### 3.3.2. Structural Feature-Based Biomass Estimation

Structural features like canopy height metrics were also used for estimating oat biomass. In this study, canopy structural features resulted in superior estimations than spectral features (Table 10). Noticeably higher estimation accuracies were obtained from models based on structural features (R^2^ ranges from 0.61 to 0.73 and RMSE% ranges from 30.48 to 36.57%) than canopy spectral features-based models. RFR also exhibited the highest estimation accuracy for biomass with R^2^ of 0.73 and RMSE% of 30.48%. With improved R^2^ and decreased RMSE%, the use of structural features improved the estimation results for oat biomass, which is also demonstrated by general convergence pattern of the spread points towards the bisector (Figure 4). Nonetheless, the SVR model still underestimates the higher values of biomass. 

A possible explanation of the superior performance of estimation models based on structural features over those based on spectral features is that canopy structural features can provide the three-dimensional canopy information and can better reflect canopy growth and biomass. Also, canopy structural features do not suffer from asymptotic saturation, unlike spectral indices [77]. Many studies have validated the potential of structural features in biomass estimation. Bendig et al. [30] reported that canopy height derived from Crop Surface Model (CSM) is a suitable indicator of biomass in barley. In their study, they tested five different models based on canopy height and predicted biomass with R^2^ of 0.8. Similarly, a study for rice crop [80] showed good estimations of biomass from canopy height (R^2^ = 0.68–0.81). Acorsi et al. [29] also showed successful estimations of fresh and dry biomass of black oats using structural features (R^2^ ranges from 0.69 to 0.81).

Many studies have highlighted the potential of combining canopy height with VIs, rather than using them separately [7,31,40,81], which resulted in robust and improved estimations of biomass yield in previous studies. Consistent with previous studies, the results in this work also show that a combination of spectral and structural features resulted in more improved estimation accuracy than using spectral or structural features alone, with R^2^ ranging from 0.66 to 0.79 and RMSE% ranging from 26.97 to 34.12% (Table 10). Canopy structural features can provide information about canopy architecture, not provided by spectral features and can, to some extent, overcome the saturation problem of spectral features. This is observed in our study as well. A combination of spectral and structural features has resolved the underestimation trend of biomass samples at higher values to some extent, which is demonstrated by scatter plots of measured vs. predicted oat biomass yield (Figure 4).

#### 3.3.3. Textural Feature-Based Biomass Estimation

Textural features have been widely used for image classification purposes and for forest biomass estimation. Textural features have also been tested in a few studies for crop biomass estimation in recent years. In these studies, textural features are often used alone [71] or in conjunction with spectral features [48,49,52]. Our study demonstrates that biomass estimation using textural features alone shows a higher accuracy than either spectral or structural features, with an R^2^ ranging from 0.834 to 0.92 and RMSE% from 16.7 to 24.02%. (Table 10). The data points are also more converged towards the bisector, which demonstrated the improved estimation performance of canopy textural feature-based biomass estimation (Figure 4). Textural features are based on spectral bands, and, thus, they show some collinearity with the spectral features. However, in contrast to spectral features, textural features can characterize canopy architecture and structure patterns to some extent [82], as well as weaken saturation issues and suppress the soil background effect. This could be a possible explanation for the superior performance of textural features over spectral features. The superior performance of textural features over spectral features in this study is in agreement with previous findings estimating forest biomass [71,83]. 

Several studies have also combined canopy spectral and textural features to improve estimation accuracy for crop biomass estimation. Wengert et al. [84] found that GLCM-based textural features improved the estimation of barley dry biomass and leaf area index. Similar results were also documented in above-ground biomass estimation of legume grass mixtures [41], rice [48], and winter wheat [52]. It is worth noting that, in this study, the estimation accuracy provided by using only textural features is higher than combining spectral and structural features. A possible explanation for the improved estimation accuracy is that textural features takes into account the spatial variation in the pixels and provides additional information about the physical structure of the canopy, edges of a canopy, and overall canopy architecture [85,86]. 

#### 3.3.4. Data Fusion and Biomass Estimation

Inclusion of textural features to the spectral and structural features provided superior estimations of oat biomass. For all three regression models, fusion of all three types of features have yielded improved estimations of biomass over using a single type of features or combining two types of features, with R^2^ varying from 0.85 to 0.92 and RMSE% ranging from 15.97% to 22.63%. However, the estimation accuracy was not substantially improved when combining all three features compared to using textural features only (Table 10). Also, it is noteworthy that the estimation accuracy provided by PLSR when using textural features is slightly higher than that provided by SVR when combining all three features. This could be associated with information overlapping and redundancy issues linked with canopy spectral, structural and textural features [77]. 

The improved performance of combination of all three features can also be observed from the scatter plots of measured vs. predicted biomass (Figure 4). Observing the distribution of data points around the bisector (black dashed line), it is found that combining all three features significantly improved the estimation accuracy as data points are more converged towards the bisector. However, what is prominent and consistent with all the three models is the underestimation of higher biomass values. The higher values of biomass are underestimated to some extent by all three models. This is likely attributed to an optical saturation issue. Overall, combining structural features with spectral features increased R^2^ by 5–14%, whereas combining all three features led to an almost 28–56% increase in R^2^, depending on the models.

Several studies have tested the combination of multiple information, by integrating non-spectral features (3D, thermal) or textural features with spectral features for the evaluation of biomass in grasslands [26,87,88] and in cultivated crops. There have been many studies that have combined spectral and textural features [48,49] or spectral and structural features [40,89] to improve biomass estimation accuracy in agricultural crops. A study conducted in soybean [12] highlighted the benefits of fusing multiple features and multi-source information for the estimation of grain yield (R^2^ = 0.72). Future research should focus more on evaluating the benefits of feature fusion (spectral, structural, thermal, textural features) and multi-source (RGB, hyperspectral, LiDAR) data fusion for oat biomass estimation purposes.

### 3.4. Performance of Different ML Models

**Figure 5:**
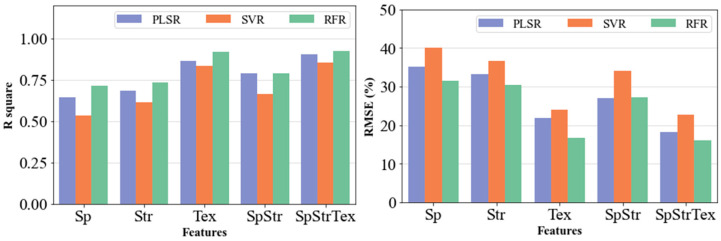
Oat biomass yields estimation performance of different models with various input feature types and numbers.

Figure 5 shows the performance of each model in the estimation of biomass based on five different input feature combinations. Based on R^2^ and RMSE%, the RFR model yielded superior performance compared to the other two models for oat biomass estimation, irrespective of the input features. In all five combinations, SVR yielded estimations with the lowest R^2^ and highest RMSE% with the poorest estimation obtained when using spectral features only as the independent variable (Figure 4 and Table 10). With all the five different input feature scenarios, RFR and PLSR generally exhibited very close performance with RFR performing slightly better. The best performance was observed when using the RFR model with all three features, giving an R^2^ of 0.92 and RMSE% of 15.97%.

Random forest is a tree-based ensemble learning method, which combines multiple predictors by building a complex non-linear relationship to solve complex problems [90]. It has gained considerable attention in terms of crop biomass modelling as it offers the advantage of faster training time, improved accuracy, higher stability, and robustness. Our results are consistent with many other studies that have demonstrated the superiority of RFR in modelling biomass and yield-related variables [26,41,84,91].

### 3.5. Limitations and Future Work

The digital terrain model used in our study has notable limitations. To obtain DTM, we employed an Inverse Distance Weighting (IDW) approach due to the absence of bare ground imagery before crop emergence. The DTM generated through this method may suffer from inherent errors, including issues related to shadows, interpolation, and the identification of ground versus off-ground surfaces, potentially resulting in underestimated canopy height data [92]. Therefore, we recommend acquiring imagery of bare ground before crop emergence to improve the reliability of DTM data. 

In terms of future work, we propose several directions. Firstly, the use of transfer learning techniques holds significant potential for enhancing model generalizability, especially when predicting biomass across different years and locations [93]. A valuable approach involves utilizing one location for training and another for testing, or likewise, one year for training and another for testing. This allows for the assessment of spatial and temporal model transferability, which is of substantial value for future research. In the realm of biomass estimation, it is important to recognize that factors beyond imagery, such as soil properties, rainfall, irrigation, weather conditions, and solar elevation angle, play a significant role. Integrating weather, soil, and crop management information could help enhance the precision and reliability of oat biomass estimations. 

## 4. Conclusions

This study investigated the potential of UAV-derived spectral, structural, textural features, and their combination for predicting the biomass yield of oats. The results show that a combination of multi-features can be superior to using spectral features alone for predicting oat biomass. The importance of canopy structural features when estimating plant biomass was highlighted by the strong relationships observed between biomass and UAV-derived canopy height. Canopy textural features also proved to be an important indicator for oat biomass estimation as the model using canopy textural features achieved higher estimation accuracy than models using spectral or structural features alone. Canopy structural and textural features likely provide a more accurate measurement of canopy architecture than spectral features and may also provide a means to overcome saturation issues associated with spectral features. All three machine-learning algorithms used in this study were highly efficient in oat biomass estimation, with RFR producing slightly higher estimation accuracies. 

In light of these findings, it is evident that the integration of multi-feature data sources, including spectral, structural, and textural features, offers a promising avenue for accurate oat biomass estimation. The underestimation trend observed for higher biomass values suggests the need for further research in addressing the optical saturation issue. Additionally, future investigations in this field should explore the advantages of incorporating additional variables, such as soil properties, weather conditions, and crop management information, to enhance the robustness and precision of oat biomass predictions. This study contributes to the growing body of research on precision agriculture and remote sensing applications, and it paves the way for more comprehensive and reliable oat biomass estimation techniques in the years to come.

## Figures and Tables

**Table 1:** Planting and harvesting dates for the forage oat variety trial conducted at South Shore and Volga in 2020 and 2021.

Year	Volga				South Shore			
	Planting	Booting Stage	Heading Stage	Milk Stage	Planting	Booting Stage	Heading Stage	Milk Stage
2020	19 May	9 July	16 July	22 July	22 May	9 July	16 July	22 July
2021	13 April	13 June	21 June	27 June	22 April	13 June	21 June	27 June

**Table 2:** Center wavelength and full width at half maximum (FWHM) bandwidth of each spectral band of Sentera Double 4K multispectral sensor.

Spectral Band	Center Wavelength (nm)	Bandwidth FWHM (nm)
Blue	446	60
Green	548	45
Red	650	70
Red Edge	720	40
Near Infrared	840	20

**Table 3:** Details of spectral features used in this study to predict biomass in oats.

S.N.	Spectral Indices	Source	Abbreviation
1	Normalized Difference Vegetation Index	[60]	NDVI
2	Green NDVI	[61]	GNDVI
3	Normalized Difference Red Edge Index	[62]	NDRE
4	Soil Adjusted Vegetation Index	[63]	SAVI
5	Optimized SAVI	[64]	OSAVI
6	Difference Vegetation Index	[65]	DVI
7	Ratio Vegetation Index	[66,67]	RVI
8	Normalized Difference Index	[66]	NDI
9	Green Leaf Index	[68]	GLI
10	Excess Green minus Excess Red Index	[69]	ExGR

**Table 4:** Digital photogrammetry-based point cloud-derived canopy structural features used in this study to predict biomass in oats.

S.N.	Height Measures	Name
1	Mean height	H_mean_
2	Median height	H_median_
3	Minimum height	H_min_
4	Maximum height	H_max_
5	Standard deviation height	H_std_
6	90th percentile	H_p90_
7	93rd percentile	H_p93_
8	95th percentile	H_p95_
9	98th percentile	H_p98_
10	99th percentile	H_p99_

**Table 5:** The grey level co-occurrence matrix (GLCM) textural features and their definitions used in this study to predict biomass in oats.

S.N.	Texture Measures	Formula
1.	Mean (ME)	$ME=\sum_{x=0}^{N-1}\sum_{z=0}^{N-1}kP(x,z)$
2.	Variance (VAR)	$VAR=\sum_{x=0}^{N-1}\sum_{z=0}^{N-1}{\left(x-\mu \right)}^{2}P(x,z)$
3.	Homogeneity (HOM)	$HOM=\sum_{x=0}^{N-1}\sum_{z=0}^{N-1}\frac{1}{{1+\left(x-z\right)}^{2}}P(x,z)$
4.	Contrast (CON)	$CON=\sum_{x=0}^{N-1}\sum_{z=0}^{N-1}{\left(x-z\right)}^{2}P(x,z)$
5.	Dissimilarity (DIS)	$DIS=\sum_{x=0}^{N-1}\sum_{z=0}^{N-1}P(x,z)\left|x-z\right|$
6.	Entropy (ENT)	$ENT=-\sum_{x=0}^{N-1}\sum_{z=0}^{N-1}P(x,z)\log\left(P\left(x,z\right)\right)$
7.	Angular Second Moment (ASM)	$ASM=\sum_{x=0}^{N-1}\sum_{z=0}^{N-1}{\left(P\left(x,z\right)\right)}^{2}$
8.	Correlation (COR)	$COR=\sum_{x=0}^{N-1}\sum_{z=0}^{N-1}P(x,z)\left[\frac{\left(x-ME)(z-ME\right)}{\sqrt{{VA}_{x}{VA}_{z}}}\right]$

Note: *P(x,z) = V(x,z)*/$\sum_{i=0}^{N-1}\sum_{j=0}^{N-1}V(x,z)$; where *V(x,z)* represents the value in the row at the cell x, and column z within the moving window. *N* represents the number of rows or columns in the window.

**Table 6:** Statistics of ground-truth oat biomass data (kg/ha) at different oat growth stages.

Year	Location	Growth Stage	No.	Mean	Min	Max.	SD
	Volga	Booting	36	7836.9	5859.3	10,076.5	1046
		Heading	36	10,934.1	6946.0	13,894.6	1514
		Milk	24	12,140.9	9931.1	13,720.9	1031.6
2020	South Shore	Booting	36	12,232.3	7789.9	15,772.4	2196.5
		Heading	36	16,418.5	13,799.2	19,645.6	1380.2
		Milk	24	17,563.1	15,187.6	20,415.8	1414.1
	All		192	14,067	5859.3	20,415.8	4116.4
		Booting	36	3754.5	2201.4	4872.8	600.3
	Volga	Heading	36	5750.66	3967.1	7287.2	713.5
		Milk	24	7873.39	6318.4	9068.8	778.8
2021	South Shore	Booting	36	3707.7	2410.1	4581.7	559.6
		Heading	36	5344.8	4002.1	7738.0	680.2
		Milk	24	5525.2	4554.4	6258.0	425.2
	All		192	5154.3	2201.4	9068.8	1476.5

**Table 7:** Pearson correlation coefficient (r) of manually measured canopy height with UAV-derived height features.

Height Features	Pearson’s Correlation Coefficient (r)
H_mean_	0.74 ***
H_median_	0.74 ***
H_min_	0.53 ***
H_max_	0.63 ***
H_std_	0.43 ***
H_p90_	0.77 ***
H_p93_	0.77 ***
H_p95_	0.77 ***
H_p98_	0.76 ***
H_p99_	0.74 ***

*p*-value significance: *** = *p* ≤ 0.001.

**Table 8:** Pearson correlation coefficient (r) of oat biomass with VIs and canopy height features.

VIs	r	Height Features	r
NDVI	0.33 ***	H_mean_	0.59 ***
NDRE	0.49 ***	H_median_	0.73 ***
GNDVI	0.24 ***	H_min_	−0.31 ***
RVI	0.37 ***	H_max_	0.61 ***
DVI	0.17 ***	H_std_	0.39 ***
SAVI	0.33 ***	H_p90_	0.74 ***
OSAVI	0.33 ***	H_p93_	0.73 ***
NDI	0.38 ***	H_p95_	0.73 ***
GLI	0.63 ***	H_p98_	0.73 ***
ExGR	0.19 ***	H_p99_	0.72 ***

*p*-value significance: *** = *p* ≤ 0.001.

**Table 9:** Pearson correlation coefficient (r) of oat biomass with textural features.

Textural Features	r	Textural Features	r
Red ME	−0.14 ***	Blue DIS	−0.07
Red VA	−0.05	Blue ENT	−0.15 ***
Red HO	−0.12	Blue ASM	−0.02
Red CO	0.01	Blue COR	−0.45 ***
Red DI	0.07	NIR ME	−0.16 ***
Red EN	−0.11 ***	NIR VAR	−0.3 ***
Red SM	0.02	NIR HOM	0.05
Red CC	−0.36 ***	NIR CON	−0.19 ***
Green ME	−0.11 ***	NIR DIS	−0.1 **
Green VA	−0.19 ***	NIR ENT	−0.14 ***
Green HO	−0.03	NIR ASM	−0.01
Green CO	−0.07	NIR COR	−0.71 ***
Green DI	−0.01	Red edge ME	−0.24 ***
Green EN	−0.27 **	Red edge VAR	−0.31 ***
Green SM	−0.15	Red edge HOM	0.11
Green CC	−0.62 ***	Red edge CON	−0.22 ***
Blue ME	−0.16 ***	Red edge DIS	−0.17 ***
Blue VA	−0.11 ***	Red edge ENT	−0.19 ***
Blue HO	0.06	Red edge ASM	−0.04
Blue CO	−0.06	Red edge COR	−0.56 ***

*p*-value significance: ** = *p* ≤ 0.01, *** = *p* ≤ 0.001.

**Table 10:** Validation statistics of oat biomass yield estimation using three machine learning methods.

Input Features	No. of Features	Metrices	PLSR	SVR	RFR
		R^2^	0.645	0.535	0.713
Spectral	10	RMSE	3325.62	3803.1	2986.49
		RMSE%	35.1%	40.14%	31.52%
		R^2^	0.682	0.614	0.732
Structural	10	RMSE	3146.57	3464.92	2887.85
		RMSE%	33.21%	36.57%	30.48%
		R^2^	0.862	0.834	0.92
Textural	24	RMSE	2068.59	2276.1	1582.69
		RMSE%	21.83%	24.02%	16.7%
Spectral + Structural		R^2^	0.79	0.664	0.788
	20	RMSE	2555.28	3233.34	2569.01
		RMSE%	26.97%	34.12%	27.11%
Spectral + Structural + Textural		R^2^	0.903	0.852	0.926
	44	RMSE	1733.61	2144.09	1512.77
		RMSE%	18.30%	22.63%	15.97%

## Data Availability

The data presented in the study will be available from the corresponding author upon request.
